# Utility of Covered Self-Expanding Metal Stents for Biliary Drainage during Neoadjuvant Chemotherapy in Patients with Borderline Resectable Pancreatic Cancer

**DOI:** 10.3390/jcm12196245

**Published:** 2023-09-28

**Authors:** Masaru Furukawa, Yasutaka Ishii, Masahiro Serikawa, Tomofumi Tsuboi, Yumiko Tatsukawa, Tetsuro Hirano, Shinya Nakamura, Juri Ikemoto, Yusuke Kiyoshita, Sho Saeki, Yosuke Tamura, Sayaka Miyamoto, Kazuki Nakamura, Yumiko Yamashita, Noriaki Iijima, Kenichiro Uemura, Shiro Oka

**Affiliations:** 1 Department of Gastroenterology, Graduate School of Biomedical and Health Sciences, Hiroshima University, Hiroshima 734-8551, Japan; mfurukawa@hiroshima-u.ac.jp (M.F.);; 2 Department of Surgery, Graduate School of Biomedical and Health Sciences, Hiroshima University, Hiroshima 734-8551, Japan

**Keywords:** self-expanding metal stent, pancreatic cancer, neoadjuvant chemotherapy

## Abstract

Objectives: We aimed to compare the utility of covered self-expanding metal stents (CSEMSs) with that of plastic stents (PSs) for biliary drainage during neoadjuvant chemotherapy in patients with borderline resectable pancreatic cancer. Methods: Forty patients with borderline resectable pancreatic cancer underwent biliary stenting during neoadjuvant chemotherapy at Hiroshima University Hospital. PSs and CSEMSs were placed in 19 and 21 patients, respectively. Two gemcitabine-based regimens for chemotherapy were used. Treatment outcomes and postoperative complications were compared between both groups. Results: The incidence of recurrent biliary obstruction was significantly lower in the CSEMS group (0% vs. 47.4%, *p* < 0.001), and the median time to recurrent biliary obstruction in the PS group was 47 days. There was no difference in the incidence of other complications such as non-occlusive cholangitis, pancreatitis, and cholecystitis between the two groups. Delays in the chemotherapy schedule due to stent-related complications were significantly frequent in the PS group (52.6% vs. 4.8%, *p* = 0.001). There was no significant difference in the incidence of postoperative complications between the two groups. Conclusions: CSEMSs may be the best choice for safely performing neoadjuvant chemotherapy for several months in patients with borderline resectable pancreatic cancer with bile duct stricture.

## 1. Introduction

Among all cancers, pancreatic cancer (PC) has the worst prognosis, with a 5-year survival rate of <10% [1,2]. In recent years, therapeutic strategies for PC have been determined based on the resectability status proposed in the National Comprehensive Cancer Network (NCCN) guideline [3]. The resectability status is classified as resectable, borderline resectable, and unresectable according to the degree of local invasion and the presence/absence of distant metastases. Among these, borderline resectable PC (BRPC) is highly likely to have a residual cancer histologically with only standard surgery [4], and in the NCCN3 and Japanese clinical practice guidelines [5], neoadjuvant therapy such as chemotherapy and chemoradiotherapy have been recommended. However, evidence to recommend specific neoadjuvant therapies is still limited, and the standard neoadjuvant regimen remains controversial.

Nearly 80% of PC are reported to occur in the pancreatic head [6], and pancreatic head cancer is often associated with obstructive jaundice. Among patients with pancreatic cancer and biliary stricture, biliary drainage is essential for patients with cholangitis or severe jaundice who are planned to undergo surgery, and for patients undergoing chemotherapy. Types of stents used for biliary drainage in patients with PC include plastic stents (PSs) and self-expanding metal stents (SEMSs). A PS is relatively inexpensive and easy to insert into the bile duct, though has a short patency period. Therefore, the use of a covered SEMS (CSEMS), with a significantly longer patency period [7] is recommended in patients with unresectable PC [3,5]. In addition, guidelines [3,5] already recommend CSEMS for biliary drainage during neoadjuvant chemotherapy. On the other hand, few reports compare the outcomes of PS and CSEMS in biliary drainage during neoadjuvant therapy in patients with BRPC [8,9,10,11,12,13,14], and there are only three reports [9,10,11] on a small number of around 20 patients limited to neoadjuvant chemotherapy. We aimed to clarify the utility of the CSEMS for biliary drainage during neoadjuvant chemotherapy in patients with BRPC.

## 2. Materials and Methods

### 2.1. Patients

This was a retrospective, single-center, observational study. Forty consecutive patients with BRPC who underwent endoscopic biliary stenting (EBS) during neoadjuvant chemotherapy at Hiroshima University Hospital from January 2010 to December 2021 were enrolled. BRPC was defined according to the NCCN guideline [3], i.e., pancreatic head tumors which contact with the superior mesenteric artery at an angle ≤ 180° or contact with common hepatic artery without extension to the celiac artery or hepatic artery bifurcation, and pancreatic body/tail tumors which contact with the celiac artery at an angle ≤ 180° are defined as BRPC-A; tumors which contact with the superior mesenteric vein or portal vein at an angle > 180° are defined as BRPC-PV. Assessment of the resectability status was performed with contrast-enhanced multidetector-row computed tomography (MDCT).

Among 40 patients, 19 patients had PSs (PS group), and the other 21 had CSEMSs (CSEMS group). In the PS group, two patients in whom metastasis was detected during surgery and could not receive curative surgery were included. 

Written informed consent was obtained from all patients and their families before endoscopic retrograde cholangiopancreatography (ERCP) was performed. This study was conducted in accordance with the Declaration of Helsinki and was approved by the Hiroshima University Hospital ethics committee (approval No. E-2843). 

### 2.2. Strategy of Biliary Drainage

ERCP was performed using a video duodenoscope (JF-260V or TJF-260V; Olympus Medical Systems, Tokyo, Japan). At our institution, endoscopic nasobiliary drainage (ENBD) was primarily performed first in patients with suspected PC who had jaundice or elevated levels of hepatobiliary enzymes to prevent the occurrence of retrograde cholangitis until the diagnosis was defined and cholecystitis due to CSEMS placement. EBS was performed after a definitive diagnosis of cancer was made by bile cytology, brush cytology, or endoscopic ultrasound-guided fine needle aspiration. However, several patients were referred to our institution after biliary stenting was performed as the first biliary drainage method at other institutions. Until May 2013, PSs were mainly selected, whereas after June 2013, CSEMSs were mainly selected. The PSs used were 7 Fr Flexima^TM^ Biliary Stent (Boston Scientific Corp., Marlborough, MA, USA), Through & Pass^®^ (GADELIUS MEDICAL, Tokyo, Japan), and Zimmon^®^ Biliary Stent (COOK MEDICAL, Bloomington, IN, USA), and the CSEMSs used were an 8 or 10 mm WallFlex^TM^ Biliary RX Stent (Boston Scientific Corp.), HANAROSTENT^®^ Biliary Full Cover (Boston Scientific Corp.), BONASTENT^®^ Biliary (Medico’s Hirata, Osaka, Japan), and Niti-S SUPREMO (TaeWoong Medical Co., Gimpo, Republic of Korea). A small endoscopic sphincterotomy (EST) with an incision range of a few millimeters was performed before stenting to prevent post-ERCP pancreatitis. All ERCP-related procedures were performed under conscious sedation of the patient with intravenous administration of midazolam alone or midazolam plus pentazocine.

### 2.3. Neoadjuvant Chemotherapy

Neoadjuvant chemotherapy was initiated after serum total bilirubin level had decreased below 2.0 mg/dL, and aspartate aminotransferase and alanine aminotransferase had decreased below 100 U/L. In this study, two neoadjuvant chemotherapy regimens were used: gemcitabine plus S-1 (GS) chemotherapy [15], and gemcitabine, nab-paclitaxel plus S-1 (GAS) chemotherapy [16]. In GS chemotherapy, patients were administered 1000 mg/m^2^ gemcitabine on days 1 and 8 and 65 mg/m^2^ S-1 on days 1–14 through 14 of a 21-day cycle, and in GAS chemotherapy, patients were administered 1000 mg/m^2^ gemcitabine on day 1, 125 mg/m^2^ nab-paclitaxel on day 1 and 60–100 mg/day S-1 on days 1–7 of a 14-day cycle. GS chemotherapy was introduced for patients with BRPC-A until January 2016. GAS chemotherapy was introduced for patients with BRPC-A in a clinical trial after February 2016 and also for patients with BRPC-PV after January 2019. Patients received three cycles of GS chemotherapy or six cycles of GAS chemotherapy and were then evaluated for resectability with MDCT. Surgical resection was performed 1–2 weeks or 2–6 weeks after completion of GS and GAS chemotherapy, respectively.

### 2.4. Outcomes

The primary outcome measure was the rate of recurrent biliary obstruction (RBO) in the PS and CSEMS groups. The secondary outcomes were the time to RBO (TRBO) and complications other than RBO in the two groups. RBO and TRBO were defined according to the TOKYO criteria 2014 [17], and the cause of RBO and complications other than RBO (non-occlusive cholangitis, cholecystitis, pancreatitis, and gastrointestinal bleeding) were evaluated in accordance with the same criteria. We also evaluated the clinical characteristics and postoperative complications in the two groups. Postoperative complications were evaluated using the Clavien–Dindo classification [18], with grade IIIa or higher being positive.

### 2.5. Statistical Analysis

Statistical analyses were performed using JMP (Version Pro 16.2.0; SAS Institute Inc., Tokyo, Japan). Continuous variables were compared using Wilcoxon rank sum test, and categorical values were compared using the chi-square test or Fisher’s exact test. The cumulative RBO rate was calculated using the Kaplan-Meier method and compared using the log-rank test. *p* values < 0.05 were considered to indicate statistical significance.

## 3. Results

### 3.1. Patient Characteristics

All forty patients completed the planned neoadjuvant chemotherapy regimen and underwent surgical operation. Thirty-eight patients received curative surgery, but the other two patients could not receive curative surgery because of metastases which were detected during surgery; one had a positive peritoneal lavage cytology, and the other had liver metastasis. The clinical profiles of patients are presented in Table 1. There was no significant difference in median age, sex, tumor size, tumor location, resectability classification, serological findings, and neoadjuvant chemotherapy regimen between the CSEMS and PS groups. In the CSEMS group, almost all patients underwent 10 mm CSEMS placement, and only two patients underwent 8 mm CSEMS placement. In the CSEMS group, all patients underwent ENBD before stenting. Alternatively, ten patients (52.6%) in the PS group did not undergo ENBD before stenting (*p* < 0.001). Five patients underwent pancreatic stent placement at the discretion of the attending physician, and all of them were in the PS group.

### 3.2. Complications Related to Biliary Stent Placement

Table 2 summarizes the complications related to biliary stent placement. RBO occurred in 10 of 19 patients (47.4%) in the PS group and none in the CSEMS group (*p* < 0.001). In the PS group, three patients (15.8%) had stent migration, and six patients (31.6%) had stent occlusion. All stent occlusions were due to sludge. Complications other than RBO occurred in four patients (21.1%) in the PS group and four patients (19.1%) in the CSEMS group (*p* = 1.000). Non-occlusive cholangitis occurred in four patients (21.1%) in the PS group and three patients (14.3%) in the CSEMS group (*p* = 0.689). Cholecystitis occurred in two (9.5%) patients in the CSEMS group and no patient in the PS group (*p* = 0.489). One patient in the CSEMS group had both non-occlusive cholangitis and cholecystitis. Pancreatitis and gastrointestinal bleeding did not occur in either group. Non-occlusive cholangitis was relieved by intravenous administration of antibiotics. Cholecystitis was relieved conservatively by percutaneous transhepatic gallbladder aspiration and intravenous administration of antibiotics.

**Figure 1:**
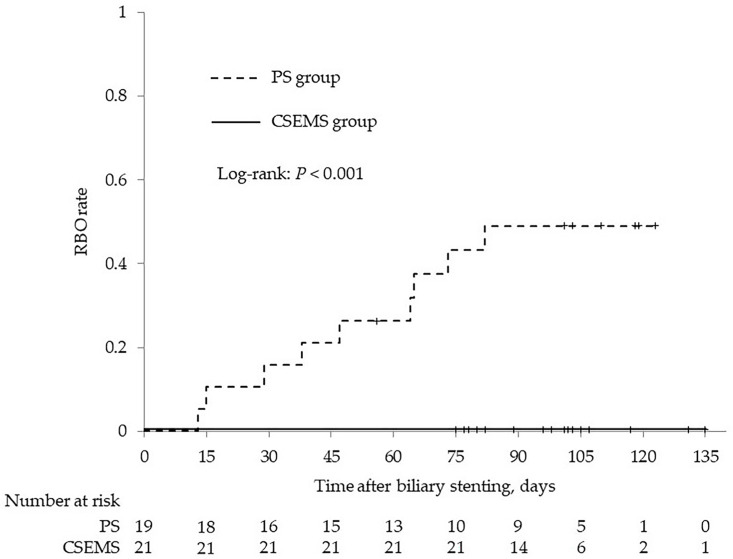
Kaplan–Meier analysis of the RBO rate. The cumulative rate was calculated using the Kaplan–Meier method and compared using the log-rank test. CSEMS, covered self-expanding metal stent; PS, plastic stent; RBO, recurrent biliary obstruction.

**Figure 2:**
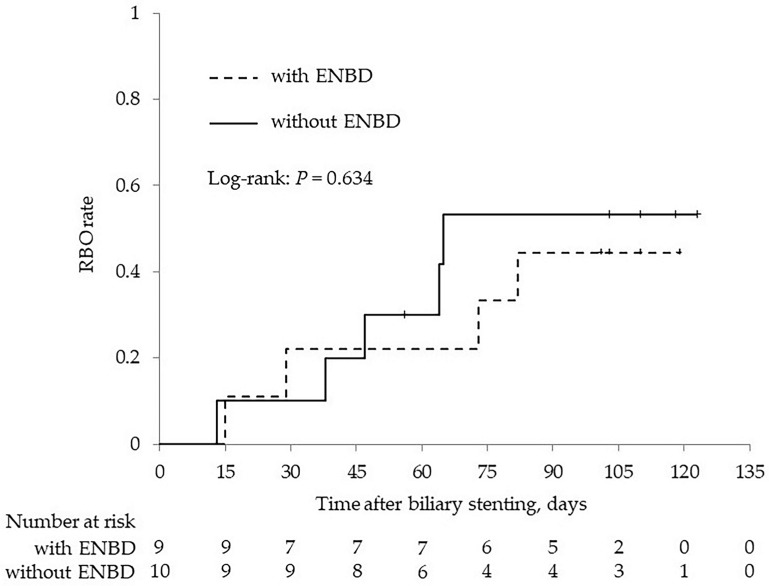
Kaplan–Meier analysis of the RBO rate with or without ENBD before stent placement in the PS group. RBO, recurrent biliary obstruction; ENBD, endoscopic nasobiliary drainage; PS, plastic stent.

Figure 1 shows the Kaplan-Meier analysis of RBO rate. The cumulative RBO rate of the PS group was 15.8% in 30 days, 26.3% in 60 days, and 49.0% in 90 days. In contrast, the cumulative RBO rate of the CSEMS group was 0% throughout 90 days, and the cumulative rate of RBO was significantly lower in the CSEMS group (log-rank: *p* < 0.001). Median TRBO of the PS group was 47 days. In the PS group, there was no significant difference in the cumulative RBO rate with or without ENBD before stent placement (Figure 2; log-rank: *p* = 0.634), and median TRBO was also not significantly different with or without ENBD (50 vs. 47 days; *p* = 0.326).

### 3.3. Clinical Course during Neoadjuvant Chemotherapy

Table 3 presents the time from initiation of neoadjuvant chemotherapy to surgery and the rate of delay in the chemotherapy schedule for each regimen. Regardless of the regimen, there was no difference in the time from the initiation of neoadjuvant chemotherapy to surgery between the CSEMS and PS groups. All delays in chemotherapy schedule were significantly more frequent in the PS group than in the CSEMS group (63.2% vs. 19.1%, *p* = 0.009). In addition, delays due to stent-related complications were also significantly more frequent in the PS group (52.6% vs. 4.8%, *p* = 0.001). Four patients in the CSEMS group had a delayed chemotherapy schedule due to non-occlusive cholangitis in one patient, strangulated small bowel obstruction in one, and leukopenia in two. On the contrary, twelve patients in the PS group had a delayed chemotherapy schedule due to biliary stent obstruction in five patients, biliary stent migration in two, both biliary stent obstruction and osteoarthritis of the hip in one, both biliary stent migration and leukopenia in one, both non-occlusive cholangitis and leukopenia in one, and leukopenia in two.

### 3.4. Outcomes of Surgery and Postoperative Complications

Table 4 shows the outcomes of surgery and postoperative complications. Potentially curative pancreatectomy was performed in 21 patients (100%) in the CSEMS group and 17 (89.5%) in the PS group. There was no difference in surgical procedures between the CSEMS group and the PS group. Serious complications of Clavien–Dindo classification grade IIIa or higher were observed in six patients (28.6%) in the CSEMS group and in five (26.3%) in the PS group; there was no significant difference between the two groups. In-hospital mortality rate was 4.8% (1/21) in the CSEMS group, and 0% (0/17) in the PS group. There was no significant difference in the length of hospital stay following surgery between the two groups.

## 4. Discussion

The present study revealed the usefulness of CSEMSs for biliary drainage during planned gemcitabine-based neoadjuvant chemotherapy in patients with BRPC. There have been several reports [8,9,10,11,12,13,14] comparing the outcomes of PS and CSEMS for biliary drainage during neoadjuvant therapy for PC (Table 5). Similar to this study, these studies showed the utility of CSEMS for PS in terms of fewer stent-related complications. However, many studies [8,10,12,13,14] also included chemoradiotherapy as neoadjuvant therapy, and only two studies [9,11] were limited to patients with BRPC who received chemotherapy alone. Although this was a retrospective study, it may provide evidence for the utility of CSEMS for biliary drainage during neoadjuvant chemotherapy in patients with BRPC. Neoadjuvant therapy for patients with BRPC has been recommended in the guidelines [3,5] to improve prognosis. The optimal neoadjuvant therapy regimen is still unclear, but several study results have been reported. The results of two randomized controlled trials for chemoradiotherapy have been reported. Jang et al. [19] reported that in the intention-to-treat analysis, median overall survival was better in the neoadjuvant chemoradiation with gemcitabine group than in the upfront surgery group (21.0 vs. 12.0 months). In the phase chemoradiotherapy has been reported. Jang et al. [19] reported that in the intention-to-treat analysis, median overall survival was better in the neoadjuvant chemoradiation with gemcitabine group than in the upfront surgery group (21.0 vs. 12.0 months). In the phase III randomized controlled trial of resectable PC and BRPC conducted in a Dutch group (PREOPANC trial) [20], there was no significant difference in the overall survival among all patients, but when limited to patients with BRPC, median overall survival was longer in the neoadjuvant chemoradiation with gemcitabine group than in the upfront surgery group (17.6 vs. 13.2 months). Regarding chemotherapy, in addition to the two regimens in this study, GS and GAS therapies, modified FOLFIRINOX therapy [21,22] and gemcitabine plus nab-paclitaxel combination therapy [22,23] have been reported to be useful in patients with BRPC, although in all single-arm studies. Randomized controlled trials comparing neoadjuvant FOLFIRINOX with gemcitabine-based chemoradiotherapy [24] and GS therapy with gemcitabine and nab-paclitaxel combination therapy [25] for resectable PC and BRPC are currently underway, and the results are awaited. It took 10–16 weeks from initiation of neoadjuvant therapy to surgery in these reports. In this study, the median time from the initiation of chemotherapy to surgery was 14 weeks for both GS and GAS therapies. Although the regimen and duration of neoadjuvant therapy have been controversial, biliary drainage using CSEMS may enable safe treatment without causing RBO in a treatment period of approximately 3 to 4 months.

Pancreatitis is one of the major complications of biliary stent placement [17]. Patients without pancreatic duct dilatation are particularly at a high risk of pancreatitis after CSEMS placement, and the usefulness of pancreatic stenting during CSEMS placement in the prevention of pancreatitis has been reported [26,27]. In this study, none of the patients in the CSEMS group developed pancreatitis following stent placement, including six patients without main pancreatic duct dilatation. Pancreatic stenting was not performed in these six patients, but all patients enrolled in this study underwent EST. There are many negative reports regarding the usefulness of EST for prevention of pancreatitis caused by CSEMS placement in patients with malignant distal biliary stricture due to pancreatic head tumors [28,29,30]. However, these studies did not consider the presence or absence of pancreatic duct dilatation, and there have been no reports on the preventive effect of EST on pancreatitis in CSEMS placement for PC without pancreatic duct dilatation. It is difficult to prove the usefulness of EST from the results of this study, so further analysis with more cases will be needed. In this study, two patients had acute cholecystitis due to obstruction of the cystic duct confluence by CSEMSs. In a retrospective cohort study of 645 patients with malignant bile duct stricture who underwent SEMS placement [31], CSEMSs were associated with significantly more cholecystitis and stent migration than uncovered SEMSs. Contrarily, a randomized trial comparing the outcomes of CSEMSs and uncovered SEMSs during neoadjuvant therapy in PC found no significant difference in the incidence of cholecystitis between the two groups [32]. There is no sufficient evidence for a therapeutic intervention method for cholecystitis caused by SEMS placement. Percutaneous transhepatic gallbladder aspiration was selected for the patients in this study, but CSEMSs can be removed and changed to PSs or short SEMS, and transpapillary gallbladder drainage is also possible. CSEMSs offers more treatment options for cholecystitis than uncovered SEMSs and may be preferable as SEMSs during neoadjuvant chemotherapy without delays in the treatment schedule.

In this study, delays in chemotherapy schedule due to stent-related complication were significantly more common in the PS group than in the CSEMS group. Fortunately, almost all RBOs in the PS group occurred during the chemotherapy withdrawal period, so there was no difference in the time from the initiation of chemotherapy to surgery compared with the CSEMS group. However, the occurrence of RBO can delay planned surgery and affect patient prognosis. 

Operation time, intraoperative blood loss, postoperative complications, and length of hospital stay did not differ between the CSEMS and PS groups in this study. In a retrospective study of 509 patients with PC who underwent pancreatoduodenectomy, SEMS did not increase postoperative complications, 30-day mortality, length of stay, but was associated with more wound infections and longer operation time [33]. It is necessary to investigate the effect of CSEMS on surgical safety in patients with BRPC who received neoadjuvant chemotherapy in a large number of cases.

This study has a few limitations. First, this was a retrospective, single-center study with small sample size, although the sample size was larger than those of other previously reported studies. However, the guidelines [3,5] already recommend CSEMSs for biliary drainage during neoadjuvant chemotherapy; therefore, it is impractical to design a prospective trial with a large sample size comparing the efficacy of CSEMSs with that of PSs. Second, there may have been a selection bias of patients due to the discretion of the attending physician on the choice of biliary stents, and it was difficult to simply compare the results, especially the relationship between cholecystitis or pancreatitis and biliary stents.

## 5. Conclusions

In conclusion, CSEMSs showed significantly lower RBO rates than PSs, and the incidence of pancreatitis and cholecystitis was similar to that in PS during 3–4 months of gemcitabine-based neoadjuvant chemotherapy in patients with BRPC. Therefore, CSEMSs may be the best biliary drainage method for safely performing neoadjuvant chemotherapy for several months in patients with BRPC.

## Figures and Tables

**Table 1:** Clinical profiles of 40 patients with BRPC undergoing biliary drainage during neoadjuvant chemotherapy.

Characteristics	CSEMS Group (*n* = 21)	PS Group (*n* = 19)	*p* Value
Median age (years)	63 (44–82)	61 (41–90)	0.456
Sex (male to female ratio)	12:9	8:11	0.527
Tumor size (mm)	30 (20–50)	30 (18–40)	0.345
Tumor location (head/body/tail)	20/1/0	19/0/0	1.000
Classification (BRPC-A/BRPC-PV)	13/8	12/7	0.935
Serological findings before drainage			
T-Bil (mg/dL)	2.7 (0.3–23.3)	5.3 (0.5–26.4)	0.704
AST (IU/L)	128 (13–818)	160 (18–321)	0.650
ALT (IU/L)	232 (12–1212)	296 (14–560)	0.884
ALP (IU/L)	1251 (84–3577)	609 (117–3857)	0.180
ENBD before stenting	21 (100)	9 (47.4)	<0.001
Neoadjuvant chemotherapy regimen			0.554
Gemcitabine plus S-1	8 (38.1)	9 (47.4)	
Gemcitabine, nab-paclitaxel plus S-1	13 (61.9)	10 (52.6)	

Data are expressed as numbers (percentages) or medians (interquartile ranges). ALP, alkaline phosphatase; ALT, alanine aminotransferase; AST, aspartate aminotransferase; BRPC, borderline resectable pancreatic cancer; BRPC-A, borderline resectable pancreatic cancer with arterial contact; BRPC-PV, borderline resectable pancreatic cancer with portal vein contact; CSEMS, covered self-expanding metal stent; ENBD, endoscopic nasobiliary drainage; PS, plastic stent; T-Bil, total bilirubin.

**Table 2:** Complications related to biliary stent placement.

	CSEMS Group (*n* = 21)	PS Group (*n* = 19)	*p* Value
Recurrent biliary obstruction	0	9 (47.4)	<0.001
Stent migration	0	3 (15.8)	0.098
Proximal side	0	1 (5.3)	0.475
Distal side	0	2 (10.5)	0.219
Stent occlusion	0	6 (31.6)	0.007
Sludge	0	6 (31.6)	0.007
Tumor ingrowth or overgrowth	0	0	-
Biliary bleeding	0	0	-
Food impaction	0	0	-
Other complications	4 (19.1)	4 (21.1)	1.000
Non-occlusive cholangitis	3 (14.3)	4 (21.1)	0.689
Cholecystitis	2 (9.5)	0	0.489
Pancreatitis	0	0	-
Gastrointestinal bleeding	0	0	-

Data are expressed as numbers (percentages). CSEMS, covered self-expanding metal stent; PS, plastic stent.

**Table 3:** Clinical course during neoadjuvant chemotherapy.

	CSEMS Group (*n* = 21)	PS Group (*n* = 19)	*p* Value
Time from the initiation of neoadjuvant chemotherapy to surgery (days)	98 (70–110)	99 (54–118)	0.407
Gemcitabine + S-1	75.5 (70–107)	87 (54–116)	0.564
Gemcitabine + nab-paclitaxel + S-1	99 (92–110)	99 (92–118)	0.412
Delay in chemotherapy schedule, *n* (%)	4/21 (19.1)	12/19 (63.2)	0.009
Gemcitabine + S-1	2/8 (25.0)	8/9 (88.9)	0.015
Gemcitabine + nab-paclitaxel + S-1	2/13 (15.4)	4/10 (40.0)	0.341
Delay in chemotherapy schedule due to stent-related complications, *n* (%)	1/21 (4.8)	10/19 (52.6)	0.001
Gemcitabine + S-1	0/8 (0)	6/9 (66.7)	0.009
Gemcitabine + nab-paclitaxel + S-1	1/13 (7.7)	4/10 (40.0)	0.127

Data are expressed as numbers (percentages) or medians (interquartile ranges). CSEMS, covered self-expanding metal stent; PS, plastic stent.

**Table 4:** Outcomes of surgical resection and postoperative complications.

	CSEMS Group (*n* = 21)	PS Group (*n* = 19)	*p* Value
Pancreatectomy	21 (100)	17 (89.5)	0.524
Surgical procedure			0.354
PD	18 (85.7)	13 (76.5)	
PD + hepatic artery resection	2 (9.5)	4 (23.5)	
TP	1 (4.8)	0	
Arterial resection	2 (9.5)	5 (29.4)	0.207
PV/SMV resection	15 (71.4)	11 (64.7)	0.734
Operation time (min)	358 (238–686)	455 (238–676)	0.081
Blood loss (mL)	819 (45–6672)	682 (158–2830)	0.965
Postoperative complications	6 (28.6)	5 (26.3)	0.955
Biliary fistula	0	0	-
Pancreatic fistula	0	2 (11.8)	0.194
Cholangitis	2 (9.5)	1 (5.9)	1.000
Gastrointestinal bleeding	0	0	-
Delayed gastric emptying	1 (4.8)	2 (11.8)	0.577
In-hospital mortality	1 (4.8)	0	1.000
Others	3 (14.3)	2 (11.8)	1.000
Length of hospital stay (days)	19 (15–285)	19 (14–63)	0.802

Data are expressed as numbers (percentages) or medians (interquartile ranges). CSEMS, self-expanding metal stent; PD, pancreatoduodenectomy; PS, plastic stent; PV/SMV, portal vein/superior mesenteric vein; TP, total pancreatectomy.

**Table 5:** Previous studies comparing the outcome of PS and CSEMS during neoadjuvant therapy for pancreatic cancer.

Study	Stents	Patients	Resectability	Neoadjuvant Therapy	RBO	Other Complications
Kubota [8], 2014	PS	21	21 BR	11 NAC (G, GS) 10 NACRT (GS)	86%	NR
	CSEMS	17	17 BR	17 NACRT (GS)	24%	NR
Tsuboi [9], 2016	PS	11	11 BR	11 NAC (GS)	63.6%	9.1%
	CSEMS	9	9 BR	9 NAC (GS)	0%	0%
Kuwatani [10], 2020	PS	12	6 R, 6 BR	6 NAC (S-1), 6 NACRT (S-1)	83%	0%
	CSEMS	17	8 R, 9 BR	8 NAC (S-1) 9 NACRT (S-1)	6%	5.8%
Tamura [11], 2021	PS	11	11 BR	11 NAC (GnP)	64%	NR
	CSEMS	11	11 BR	11 NAC (GnP)	18%	NR
Kobayashi [12], 2021	PS	22	15 R, 7 BR	22 NACRT (S-1)	95.4%	0%
	CSEMS	21	13 R, 8 BR	21 NACRT (S-1)	4.8%	4.8%
Hasegawa [13], 2021	PS	40	1 R, 32 BR, 7 UR	37 NACRT 3 NAC	97%	13%
	CSEMS	27	3 R, 19 BR, 5 UR	25 NACRT 2 NAC	15%	11%
Vehviläinen [14], 2022	PS	91	NR	NAC/NACRT	21%	NR
	CSEMS	15	NR	NAC/NACRT	3%	NR

BR, borderline resectable; CSEMS, covered self-expanding metal stent; G, gemcitabine; GnP, gemcitabine plus nab-paclitaxel; GS, gemcitabine plus S-1; NAC, neoadjuvant chemotherapy; NACRT, neoadjuvant chemoradiotherapy; NR, not reported; PS, plastic stent; R, resectable; RBO recurrent biliary obstruction; UR, unresectable.

## Data Availability

The data presented in this study are available on request from the corresponding author. The data are not publicly available due to privacy issues.
